# Long-term risk of stroke after transient ischaemic attack: a hospital-based validation of the ABCD^2^ rule

**DOI:** 10.1186/1756-0500-7-281

**Published:** 2014-05-04

**Authors:** Rose Galvin, Penka A Atanassova, Nicola Motterlini, Tom Fahey, Borislav D Dimitrov

**Affiliations:** 1 HRB Centre for Primary Care Research, Department of General Practice, Royal College of Surgeons in Ireland, 123 St. Stephens Green, Dublin 2, Republic of Ireland; 2 Clinic of Cerebrovascular Diseases, Department of Neurology, Medical University, 15A V Aprilov Blvd, Plovdiv 4000, Bulgaria; 3 Academic Unit of Primary Care and Population Sciences, University of Southampton, Southampton SO16 6YD, United Kingdom

**Keywords:** Stroke, Transient ischaemic attack, Risk prediction, ABCD^2^ rule, Bulgaria

## Abstract

**Background:**

The ABCD^2^ clinical prediction rule is a seven point summation of clinical factors independently predictive of stroke risk. The purpose of this cohort study is to validate the ABCD^2^ rule in a Bulgarian hospital up to three years after TIA.

**Methods:**

All consecutive admissions to an emergency department with symptoms of a first TIA were included. Baseline data and clinical examinations including the ABCD^2^ scores were documented by neurologists. Discrimination and calibration performance was examined using ABCD^2^ cut-off scores of ≥3, ≥4 and ≥5 points, consistent with the international guidelines. The Hosmer-Lemeshow test was used to examine calibration between the observed and expected outcomes as predicted by ABCD^2^ score within the logistic regression analysis.

**Results:**

Eighty-nine patients were enrolled to the study with a mean age of 63 years (+/- 12 years). Fifty-nine percent (n = 53) of the study population was male. Seven strokes (7 · 8%) occurred within the first year and six further strokes within the three-year follow-up period. There was no incident of stroke within the first 90 days after TIA. The rule demonstrated good predictive (OR = 1 · 58, 95% CI 1 · 09-2 · 29) and discriminative performance (AUC_ROC_ = 0 · 72, 95% CI 0 · 58-0 · 86), as well as a moderate calibration performance at three years.

**Conclusion:**

This validation of the ABCD^2^ rule in a Bulgarian hospital demonstrates that the rule has good predictive and discriminative performance at three years. The ABCD^2^ is quick to administer and may serve as a useful tool to assist clinicians in the long-term management of individuals with TIA.

## Background

**Figure 1:**
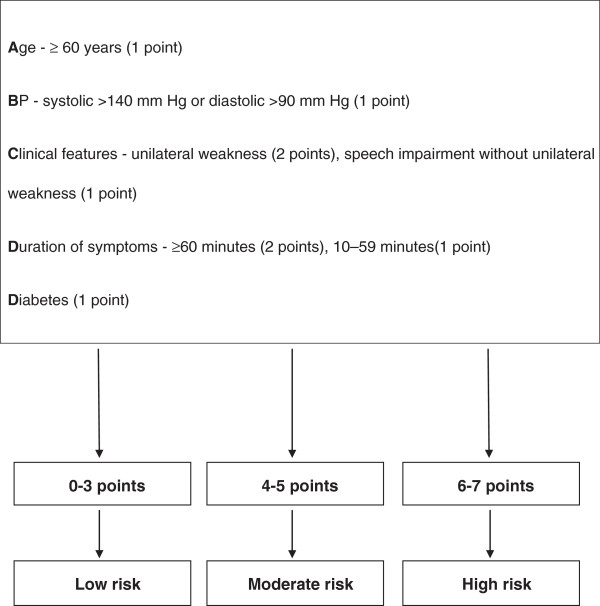
**Summary of the ABCD**^**2 **^**score.**

Transient ischaemic attack (TIA) and thrombotic stroke arise from identical aetiologies and a number of studies show that TIAs carry a significant risk of subsequent stroke [1]. A recent systematic review reported that the seven day pooled stroke risk after TIA was 6 · 2% but there was substantial heterogeneity between the primary studies included in the review with risks ranging from 0% to 18 · 7% [2]. Likewise, conflicting results have been reported in the literature on long-term risk of stroke and mortality following TIA or minor stroke in population and hospital based cohort studies [3]. Several clinical scales have been developed to improve stratification of recurrent stroke risk [4]. Identifying high-risk patients after TIA is important because timely assessment and management of these patients is essential. The ABCD^2^ clinical prediction rule (CPR) was derived in 2007 to assist clinicians with the most appropriate management of individuals with TIA [5]. The ABCD^2^ rule is a seven point summation of clinical factors independently predictive of stroke risk. These factors include age, clinical features such as motor impairment and speech disturbance, duration of symptoms, history of diabetes and hypertension. The rule identifies three strata of stroke risk after TIA; low risk (0–3 points), moderate risk (4–5 points) and high risk (6–7 points) [5]. The ABCD^2^ rule has been recommended for use in the management of patients with TIA in several international guidelines [6-9]. A summary of the rule is displayed in Figure 1.

The ABCD^2^ score was derived and validated to predict risk of stroke up to 90 days after TIA in specialist clinic cohorts and emergency departments (ED) from Oxfordshire in the UK and from California, USA [5,10]. A number of studies have validated the rule for predicting short term risk of stroke after TIA and reported inconsistent results, ranging from excellent predictive value to little better than chance, largely due to the varied methodological approaches. However, the evidence from two recent systematic reviews demonstrates that the rule has good predictive and discriminative accuracy up to 90 days after stroke [2,10]. More recently, studies have attempted to validate the rule to predict long term risk of stroke after TIA [11-13]. While these studies have reported that the rule has moderate predictive and discriminative ability, the widespread applicability of the ABCD^2^ score to predict long-term risk of stroke after TIA depends on its consistency of performance in different studies and settings [10]. Therefore, further broad validation studies by investigators from different specialties and healthcare settings are necessary. This is the first broad validation study that examines the long term predictive value of the ABCD^2^ rule in a Bulgarian cohort of patients admitted to ED after a TIA.

## Methods

### Study design and setting

The Plovdiv project is a hospital-based cohort study from 2002–2005, designed to determine predictors of incidence and prognosis of major vascular events including the recurrence of cerebrovascular or cardiovascular events. The protocol for this project is described in detail elsewhere [14,15]. Our study represents a secondary analysis of data collected from a prospective cohort of consecutive patients, presenting with symptoms of TIA to the emergency department at the University Hospital, Medical University of Plovdiv, Bulgaria. The STROBE standardized reporting guidelines were followed to report the findings of the study. Ethical approval was obtained from the Medical University of Plovdiv ethics committee.

### Study population

Patients were included in this study if they presented to the emergency department with symptoms of a first TIA, were aged >40 years and resident in Plovdiv. Consecutive TIA patients were registered prospectively. Baseline data and the clinical examinations, including those contained in the ABCD^2^ score, were documented by the study neurologists at the recruitment of patients. Determination of the ABCD^2^ risk score was performed in a manner identical to that reported by its developers. All patients underwent a CT or MRI scan to determine the index event. Patients with a clinical diagnosis other than TIA, or who were unwilling to give informed consent were excluded from the study.

### Outcomes

The TIA, as index event, was defined as a focal neurological deficit, clearly related to one of the main vascular territories, lasting from few minutes up to 24 hours and resolving completely within 24 hours, without evidence of a recent infarction on a CT scan or MRI scan of the brain [16]. Our primary outcome was the occurrence of stroke at three years after TIA. However, in-keeping with other studies of that have reported the short-term predictive value of the rule, we also examined the incidence of stroke at seven and 90 days also. A subsequent stroke was defined using the World Health Organization (WHO) definition of a clinical syndrome consisting of ‘rapidly developing clinical signs of focal (at times global) disturbance of cerebral function lasting more than 24 hours or leading to death with no apparent cause other than that of vascular origin’ and this definition was used for our study [17]. All strokes were assessed by MRI or CT scan and confirmed by a study neurologist. The determination of the outcome endpoint was done when blinded to the ABCD^2^ score and its variables. In terms of patient follow-up, evaluations were conducted by the study physicians during hospital visits every three months for the first year and then annually for 36 months. Every evaluation was carried out by contact with the patient and where relevant with the family member or caregiver.

### Statistical analysis

Descriptive statistics including means and standard deviations were computed for baseline data. We examined two aspects of validity of our results, discrimination and calibration. Discrimination refers to the ability of ABCD^2^ score to distinguish correctly the patients with different outcomes (stroke/no stroke). Logistic regression, Cox regression with cumulative hazard function, and receiver operation characteristics (ROC) curves with 95% confidence intervals (95% CI) were used for the analysis. The dependent variable was binary (stroke/no stroke) and the predictor variables examined were the range of scores on the ABCD^2^ rule. In addition, 2×2 cross-tables were used to calculate discriminative accuracy (sensitivity and specificity) at ≥3, ≥4 and ≥5 points on the ABCD^2^ rule. These different cut-points are used to identify people at low and high risk of stroke following TIA in several international guidelines on the management of patients with TIA. The *c* statistic, or area under the curve (AUC), with 95% CI were also estimated to describe model discrimination. The *c* statistic ranges from 0 · 5 (no discrimination) to a theoretical maximum of 1, values between 0 · 7 and 0 · 9 represent moderate accuracy and greater than 0 · 9 represents high accuracy [18]. A *c* statistic of 1 represents perfect discrimination, whereby scores for all cases with stroke are higher than those for all the non-cases with no overlap.

Calibration (or reliability) reflects how closely predicted outcomes agree with the actual outcomes. For this purpose, the Hosmer-Lemeshow test (HLT) was used for comparison between the observed outcomes and those expected as predicted by ABCD^2^ score within the logistic regression analysis. We also used the frequency distribution patterns in the initial derivation study of the ABCD^2^ rule as a predictive model against which our validation study was compared. Therefore the predicted number of patients with stroke at seven and 90 days (based on the probability calculated in the derivation study) was compared with the observed number of patients with stroke from our validation study. All tests were two sided. An association was considered significant at p < 0 · 05. All statistical analyses were completed using STATA (version 12, Stata Corp, College Station, Texas, USA) and IBM SPSS Statistics (Ver.21, IBM Corporation, USA).

## Results

### Demographics

From January 2002 to December 2005, a total of 98 patients with a confirmed TIA were deemed eligible for inclusion to the study. Eighty-nine patients provided written informed consent. The time from onset of symptoms to enrollment was less than 12 hours in all cases. The mean age of patients was 63 years (±12 years). Fifty-nine percent (n = 53) of the cohort was male. Baseline characteristics of enrolled patients are presented in Table 1. All patients were followed up during the study period. There were 13 subsequent strokes observed in the cohort of patients, representing an overall stroke rate of 14 · 6% at three years. There was no incident of stroke within the first seven or 90 days after TIA. Seven strokes (7 · 8%) occurred within the first year and six further strokes within the 3-year follow-up period. The stroke specific mortality rate was 4 · 5% (n = 4) and all cause mortality was 10 · 1% (n = 9) at three years.

**Table 1:** Baseline characteristics of patients with TIA

**Patient characteristics**	Parameters*
Age (years)	63 (±12)
Gender (male)	53 (59.5%)
Systolic BP (mmHg)	152 (±23)
Diastolic BP(mmHg)	91 (±15)
Hypertension	66 (74.2%)
Clinical features	
*Unilateral weakness*	43 (48.3%)
*Speech disturbance without weakness*	10 (11.2%)
Duration of symptoms (minutes)	
*≥60*	29 (32.6%)
*10-59*	44 (49.4%)
*<10*	16 (18%)
Diabetes	19 (21.3%)
Time from onset to enrollment (hours)	< 12
History of:	
*Smoking*	38 (42.7%)
*Atrial fibrillation*	2 (2.2%)
*Angina*	15 (16.9%)
*High cholesterol*	6 (6.7%)
*Anti-platelet therapy*	6 (6.7%)
*Statins*	0 (0%)

*Note: Mean (±*SD) or number (percent), as appropriate.*

### Discrimination

**Figure 2:**
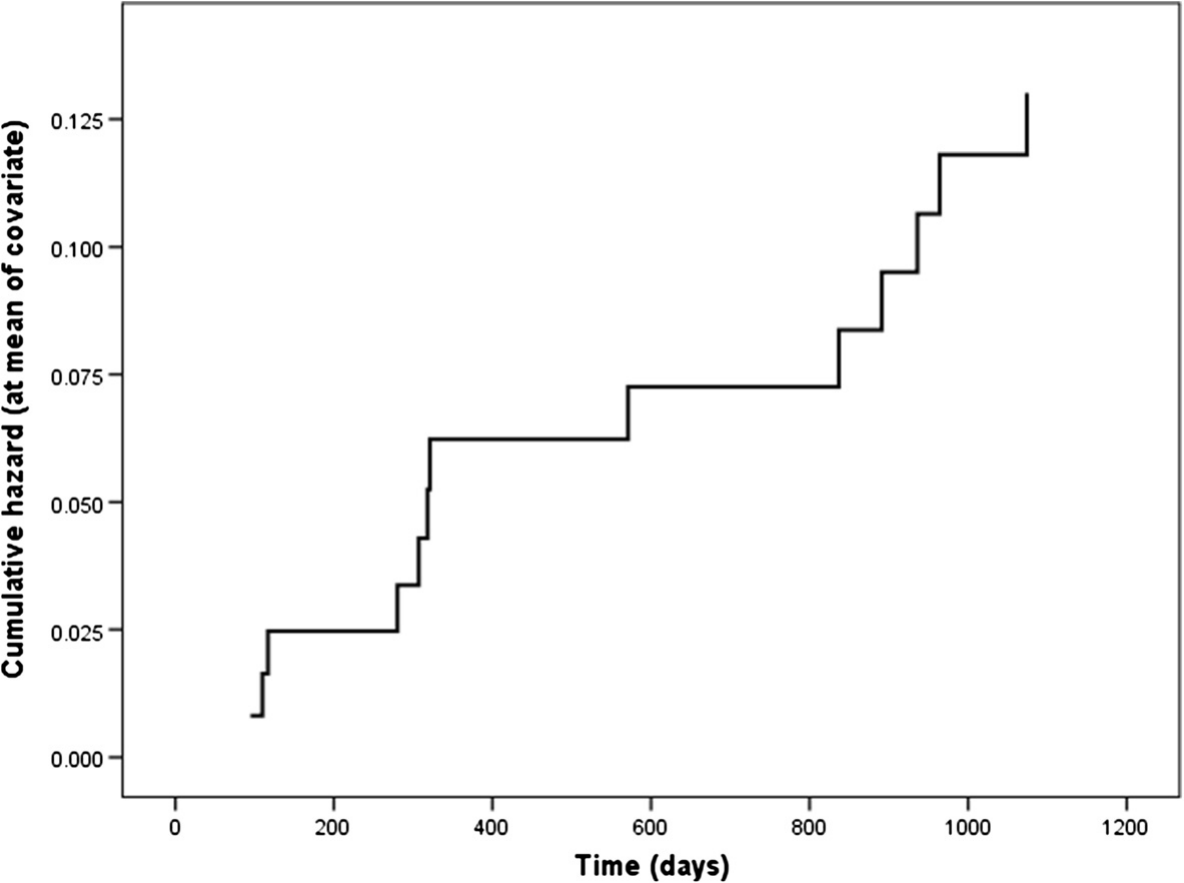
Cumulative curve illustrating the increasing risk of stroke within 3 years of TIA.

The one year and three year incidence of stroke stratified according to ABCD^2^ score (≥3, ≥4 and ≥5 points) is presented in Table 2. The logistic regression analysis indicates that the ABCD^2^ score is a significant predictor of stroke at one year (OR = 1 · 79, 95% CI 1 · 05-3 · 08, p = 0 · 03) and three years (OR = 1 · 58, 95% CI 1 · 09-2 · 29, p = 0 · 02), indicating that on average, a one point increase in the ABCD^2^ score may lead to a 58% increase in the probability of stroke at three years. Its time-independence as a significant predictor is further confirmed by the Cox regression model showing that the baseline cumulative hazard of 0 · 02 is expected to increase to 0 · 13 when the mean ABCD^2^ score is taken into account (Figure 2). The hazard function provides narrower, time-adjusted estimates of the confidence interval of the expected stroke risk of 1 · 57, from 1 · 12 to 2 · 21.

**Table 2:** The incidence of stroke up to three years stratified according to the ABCD2 score

**Variable**	**ABCD2 score**							
	**0**	**1**	**2**	**3**	**4**	**5**	**6**	**7**
**All patients (n = 89)**	4	8	16	12	17	12	15	5
**Stroke within 7 days**	0	0	0	0	0	0	0	0
**Stroke within 90 days**	0	0	0	0	0	0	0	0
**Stroke within 1 year**	0	0	0	1	1	1	3	1
**Stroke within 3 years**	0	0	1	1	1	0	3	0

**Figure 3:**
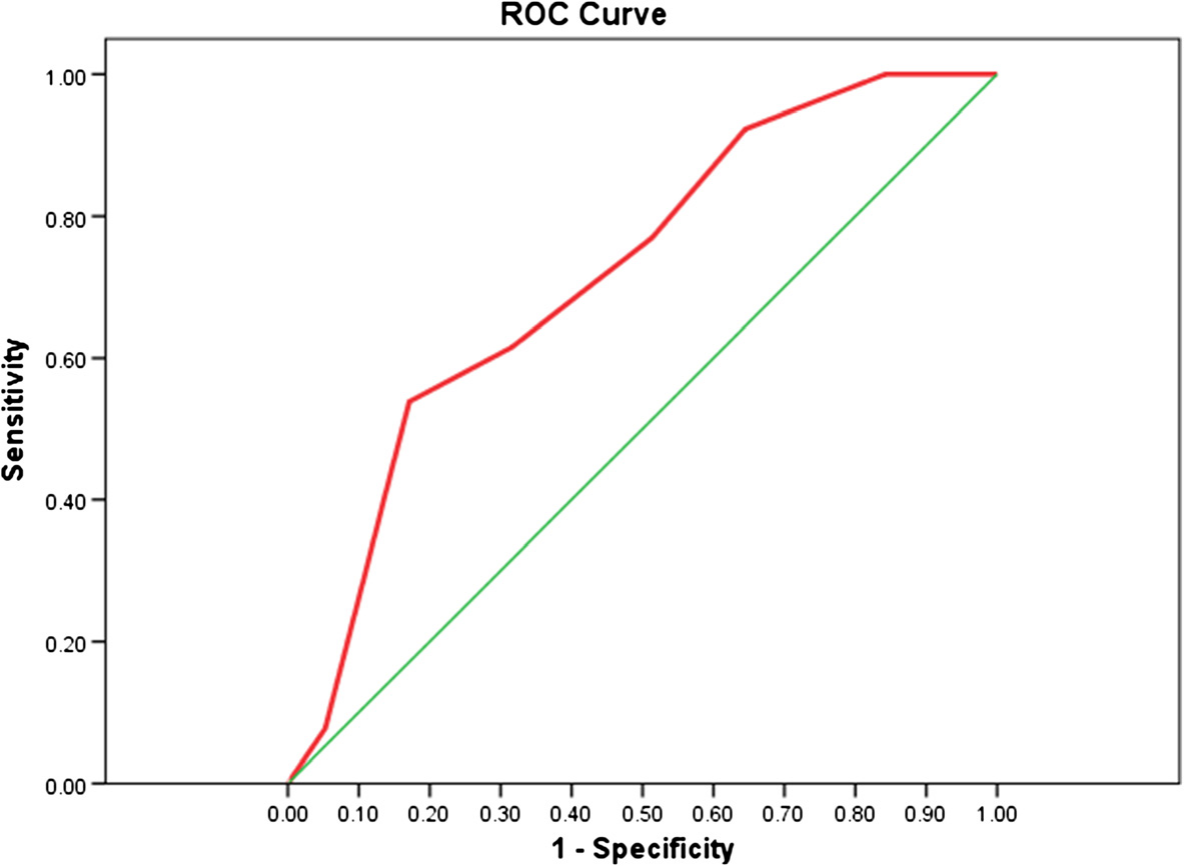
**Discriminative performance of ABCD**^**2**^** score within 3 years of TIA.**

The overall discriminative performance of the ABCD^2^ rule at three years is also confirmed by the ROC curve analysis (Figure 3) indicating about 72% overall accuracy by a significant area under the curve at p < 0 · 013 (AUC_ROC_ = 0 · 72, 95% CI 0 · 58-0 · 86). Higher estimates of discriminative performance were found at one year (AUC_ROC_ = 0 · 76, 95% CI 0 · 66-0 · 85). The specific indicators of discriminative performance at one and three years are displayed as summary estimates of sensitivity and specificity and AUCs for the different cut-off scores (Table 3). At one year following stroke, the ABCD^2^ rule is more useful at ruling out stroke in those classified as low risk using all three cut-points, with a greater summary estimates of sensitivity than specificity. The results are broadly similar at three years.

**Table 3:** **Discriminative value of the ABCD**^**2 **^**rule to identify patients at high risk of stroke at one and three years**

**Patients at high risk of stroke (as per international guidelines)**	**Discrimination (95% confidence interval)**		
**ABCD**^**2**^ **≥ 3 (US guidelines)**	**Sensitivity**	**Specificity**	**Area under curve**
**1 year**	1.00 (0.59 - 1.00)	0.34 (0.24 - 0.45)	0.67 (0.62 – 0.72)
**3 years**	0.92 (0.64 - 1.00)	0.36 (0.25 - 0.47)	0.64 (0.55 – 0.73)
**ABCD**^**2**^ **≥ 4 (UK & New Zealand guidelines)**			
**1 year**	0.86 (0.42 - 1.00)	0.48 (0.36 - 0.59)	0.67 (0.55 – 0.76)
**3 years**	0.77 (0.46 - 0.95)	0.49 (0.37 - 0.60)	0.63 (0.50 – 0.76)
**ABCD**^**2**^ **≥ 5 (Australian guidelines)**			
**1 year**	0.71 (0.29 - 0.96)	0.67 (0.56 - 0.77)	0.69 (0.51 – 0.88)
**3 years**	0.62 (0.32 - 0.86)	0.68 (0.57 - 0.79)	0.65 (0.50 – 0.80)

### Calibration

At three years, the analysis of the overall calibration performance indicates that there is no statistically significant difference between the observed and expected outcomes as predicted by the ABCD^2^ rule (*χ*^2^_HLT_ = 4.11, p = 0 · 533). When further using the original derivation study as a short-term predictive model, the ABCD^2^ rule tends to over-predict the risk of stroke at seven days across all three original risk strata: low risk, [RR 3 · 00, 95% CI (0 · 13-71 · 51)]; intermediate risk, [(RR 5 · 00, 95% CI (0 · 25-99 · 82)]; and high risk, [(RR 4 · 33, 95% CI (0 · 75-24 · 90)]. The results are broadly similar at 90 days across all three risk strata: low risk, [RR 3 · 00, 95% CI (0 · 13-71 · 51)]; intermediate risk, [(RR 5 · 00, 95% CI (0 · 25-99 · 82)]; and high risk, [(RR 9 · 00, 95% CI (0 · 52-156 · 91)]. There are seven strokes predicted at 90 days when using the original derivation study as a predictive model but no incident of stroke was observed in our dataset within this time across all three risk strata of risk.

## Discussion

### Statement of principal findings

This broad validation of the ABCD^2^ clinical prediction rule in Bulgarian patients demonstrates that the rule has a good predictive and discriminative performance, as well as a moderate calibration performance at three years. The results also indicate that the rule over-predicts the risk of stroke at seven and 90 days in comparison with the frequency distributions patterns from the original derivation study.

### Results in the context of the current literature

Our findings are broadly in-keeping with two other retrospective studies that have examined the long term predictive value of the ABCD^2^ rule [11,12]. In our study, 7 · 8% of patients had a stroke within the first year and six further strokes occurred within the three year follow-up period (14 · 94%). These results are similar to a German multi-centre hospital based validation of the ABCD^2^ rule where the overall risk of stroke at one year was estimated at 6 · 5% in a cohort of 1448 patients [3]. Our findings are also in keeping with a retrospective study by Harrison and colleagues who, although not showing results at three years, had reported that the overall absolute risk of stroke in patients who attended an outpatient clinic with a TIA was 7 · 3% within one year, 16 · 2% within five years and 28 · 0% within ten years [12]. Similar to our study, patients with higher ABCD^2^ scores (≥3 points) experienced higher stroke risk within one year and this risk persisted at five and ten years [12].

A recent systematic review of studies that validated the ABCD^2^ rule reported a low overall rate of stroke at seven days (1 · 72%) and 90 days (2 · 63%) in patients identified as low risk using an ABCD^2^ score of 0–3 points [2]. Furthermore, these patients represented one third of the overall cohort of patients in the meta-analysis [2]. Almost 45% of individuals in our dataset were classified as low risk using the ABCD^2^ cut-off score and there was no incident of stroke in this patient group within seven or 90 days. This finding in not surprising given the low stroke rate reported in the pooled analysis of studies. In addition, our results are similar to three other hospital based validation studies where no event of stroke was recorded in the low risk group at seven days [19-21]. While we reported no incident of stroke across all three strata of risk at seven or 90 days, a study by Coutts and colleagues also reported no incident of stroke across all three risk groups at seven or 90 days in a cohort of 87 patients [20]. However, our results need to be interpreted with caution due to the large number of patients in the low risk category and limited sampled population included in the validation study.

The overall discriminative value of the ABCD^2^ score at three years is significantly better than chance, as measured by ROC area under the curve and associated 95% CIs being above the null value of 0 · 5. While no other study has examined the discriminative performance of the rule at three years, the accuracy of the rule has been examined up to 13 · 8 years and an acceptable level of accuracy by AUC of 0 · 630 (95% CI 0 · 58-0 · 67) was reported [12]. Our results are also similar to those reported in a systematic review of the discriminative accuracy of the ABCD^2^ at seven days [AUC 0 · 72 (95% CI 0 · 63-0 · 82)] [10], suggesting that the rule has reasonable ability to correctly distinguish patients with and without stroke at three years.

### Strengths and weaknesses of the study

This is the first broad hospital based validation study to examine the predictive and discriminative value of the ABCD^2^ score in a Bulgarian cohort up to three years after TIA. The risk of stroke is presented in risk strata consistent with those applied in the international guidelines on the management of stroke so that the value of the ABCD^2^ score across these strata can be interpreted in a clinically meaningful way. Furthermore, the ABCD^2^ rule was applied to our cohort of patients according to the methods described by original developers [5]. Our method of calibration analysis further examines the specific performance ability of the rule by using the ratio of predicted stroke (from the original derivation study) to observed stroke in the present validation study. However, the generalisability of our results may be influenced to some extent by the relatively limited number of the patients included in our study. The comparability of our findings to the current literature is restricted by the limited number of studies that examine the long-term discriminative performance of the ABCD^2^. Therefore there is a need for future large multi-centre cohort studies to examine the long term predictive value of the rule in different clinical settings. The impact of the rule in the ED setting also warrants further investigation, particularly in terms of patient outcome, clinician behaviour, cost effectiveness and resource use, or any combination of these.

### Clinical and policy implications

Current international guidelines recommend that individuals with low ABCD^2^ scores should be triaged for specialist assessment within one week of onset of symptoms. While we observed no incident of stroke in our patients with ABCD^2^ score of 0–3 points at seven days, it is worth noting that the overall stroke rate in these patients was 7 · 5% within three years. Therefore, even at a lower risk initially, there is a need for a regular review of these specific patients in the primary care or specialist setting to monitor and manage their ongoing cerebrovascular risk and minimise the risk of subsequent stroke or other related cardiovascular events.

There is a lack of consistency in the international guidelines on what constitutes a ‘low’ and ‘high’ risk patient and we have reported our data accordingly. All guidelines relating to the management of individuals classified as ‘high risk’ include early neurology consultation to confirm the diagnosis of TIA, rapid diagnostic assessment, and implementation of aetiology-specific precautionary measures, including carotid endarterectomy and anticoagulation [2]. However, there is a need for consensus in relation to the identification of individuals at high risk of subsequent stroke as the various cut-points of ABCD^2^ score used to categorise stroke risk have considerable economic and management implications.

## Conclusion

This validation of the ABCD^2^ rule in a Bulgarian hospital setting demonstrates that the rule has a good predictive and discriminative performance at three years. The results also indicate that the rule tends to over-predict the risk of stroke at seven and 90 days in comparison with the frequency distributions patterns from the original derivation study. The ABCD^2^ is easy and quick to administer and it is a useful tool to assist clinicians in the long term management of individuals with TIA.

## Competing interests

The authors declare that they have no competing interests.

## Authors’ contributions

All authors were involved in the study conception and design. PA performed the data extraction. RG, BDD and NM acquired data for analysis, performed statistical analysis and interpretation of data and drafted the paper. TF, PA and BDD critically revised the draft manuscript. All authors read and approved the final manuscript.
